# Varespladib Inhibits the Phospholipase A_2_ and Coagulopathic Activities of Venom Components from Hemotoxic Snakes

**DOI:** 10.3390/biomedicines8060165

**Published:** 2020-06-17

**Authors:** Chunfang Xie, Laura-Oana Albulescu, Kristina B. M. Still, Julien Slagboom, Yumei Zhao, Zhengjin Jiang, Govert W. Somsen, Freek J. Vonk, Nicholas R. Casewell, Jeroen Kool

**Affiliations:** 1 Amsterdam Institute of Molecular and Life Sciences, Division of BioAnalytical Chemistry, Department of Chemistry and Pharmaceutical Sciences, Faculty of Sciences, Vrije Universiteit Amsterdam, De Boelelaan 1085, 1081 HV Amsterdam, The Netherlands; c.xie@vu.nl (C.X.); k.b.m.still@vu.nl (K.B.M.S.); j.slagboom@vu.nl (J.S.); g.w.somsen@vu.nl (G.W.S.); freek.vonk@naturalis.nl (F.J.V.); 2 Centre for Analytical Sciences Amsterdam (CASA), 1098 XH Amsterdam, The Netherlands; 3 Centre for Snakebite Research and Interventions, Liverpool School of Tropical Medicine, Pembroke Place, Liverpool L3 5QA, UK; Laura-Oana.Albulescu@lstmed.ac.uk (L.-O.A.); Nicholas.Casewell@lstmed.ac.uk (N.R.C.); 4 Centre for Drugs and Diagnostics, Liverpool School of Tropical Medicine, Pembroke Place, Liverpool L3 5QA, UK; 5 Institute of Pharmaceutical Analysis, College of Pharmacy, Jinan University, Huangpu Avenue West 601, Guangzhou 510632, China; yumeir612@hotmail.com (Y.Z.); jzjjackson@hotmail.com (Z.J.); 6 Naturalis Biodiversity Center, Darwinweg 2, 2333 CR Leiden, The Netherlands

**Keywords:** varespladib, nanofractionation, PLA_2_ activity, coagulopathic toxicity, neutralization

## Abstract

Phospholipase A_2_ (PLA_2_) enzymes are important toxins found in many snake venoms, and they can exhibit a variety of toxic activities including causing hemolysis and/or anticoagulation. In this study, the inhibiting effects of the small molecule PLA_2_ inhibitor varespladib on snake venom PLA_2_s was investigated by nanofractionation analytics, which combined chromatography, mass spectrometry (MS), and bioassays. The venoms of the medically important snake species *Bothrops asper*, *Calloselasma rhodostoma*, *Deinagkistrodon acutus*, *Daboia russelii*, *Echis carinatus*, *Echis ocellatus,* and *Oxyuranus scutellatus* were separated by liquid chromatography (LC) followed by nanofractionation and interrogation of the fractions by a coagulation assay and a PLA_2_ assay. Next, we assessed the ability of varespladib to inhibit the activity of enzymatic PLA_2_s and the coagulopathic toxicities induced by fractionated snake venom toxins, and identified these bioactive venom toxins and those inhibited by varespladib by using parallel recorded LC-MS data and proteomics analysis. We demonstrated here that varespladib was not only capable of inhibiting the PLA_2_ activities of hemotoxic snake venoms, but can also effectively neutralize the coagulopathic toxicities (most profoundly anticoagulation) induced by venom toxins. While varespladib effectively inhibited PLA_2_ toxins responsible for anticoagulant effects, we also found some evidence that this inhibitory molecule can partially abrogate procoagulant venom effects caused by different toxin families. These findings further emphasize the potential clinical utility of varespladib in mitigating the toxic effects of certain snakebites.

## 1. Introduction

Phospholipases A_2_ (PLA_2_s) are key enzymes involved in many events in cellular signaling and act by cleaving ester bonds in phospholipids to generate fatty acids (hydrolysis reactions) [1,2,3]. They are pervasive in the mammalian pancreas and are highly abundant in many animal venoms [4,5]. Venom PLA_2_ enzymes show a wide variety of functional activities, and thus can contribute to several distinct pathologies in envenomed prey/people, as well as potentially helping with prey digestion [4,5]. They are recognized as the most thoroughly investigated venom toxins both in hemotoxic and neurotoxic snake venoms [6,7]. Snake venom PLA_2_s are capable of contributing to presynaptic and/or postsynaptic neurotoxicity, myotoxicity, and cardiotoxicity, which can induce platelet aggregation disorders, hemolysis, anticoagulation, convulsions, hypotension, edema, and necrosis [4,5,8]. They play an important role in contributing to the morbidity and mortality of snakebite victims, via paralysis and destruction of respiratory muscle tissues, and/or due to their effect on homeostatic mechanisms involved in coagulation and oxygen transport [9]. Although snakebite envenoming is a severe medical problem that was recently added to the World Health Organization (WHO) list of Neglected Tropical Diseases [10], it has for a long time been systematically neglected by governments worldwide, despite over 100,000 people dying annually [11]. Although current snakebite treatments, known as antivenoms (equine/ovine polyclonal antibodies), can be effective therapies capable of reducing morbidity and mortality, they have many limitations associated with them, leaving a critical therapeutic gap between snakebite and effective treatment [6,12]. Small molecule toxin inhibitor-based approaches are gaining much traction as promising alternatives and/or complementary treatments for snakebite [12,13,14,15,16], as they show a number of characteristics desirable for use as either early prehospital or adjunct therapies [13].

Varespladib is an indole-based nonspecific pan-secretory PLA_2_ (sPLA_2_) inhibitor that potently inhibits mammalian sPLA_2_-IIa, sPLA_2_-V, and sPLA_2_-X, and in addition has been shown to inhibit venom PLA_2_ toxins [17,18,19,20]. Varespladib was originally found to reduce PLA_2_ concentrations in vivo, making it a candidate treatment for several cardiovascular diseases [17,21,22], including the treatment of acute coronary syndrome and systemic inflammatory response syndrome, but it was abandoned during Phase III clinical trials due to lack of efficacy [17,23,24,25]. Recently, varespladib was repurposed for exploration as a potential therapeutic candidate for snakebite, with early findings showing that varespladib and its orally bioavailable prodrug methyl-varespladib effectively suppress venom-induced PLA_2_ activity both in vitro and in vivo [9]. Moreover, varespladib effectively reduces hemorrhage, edema, myonecrosis, and neurotoxicity in mice caused by venoms of several medically important snakes, and as such is a potential prereferral drug candidate for treating snakebites [25,26,27,28]. In addition to varespladib, the orally available prodrug methyl-varespladib is effective in inhibiting neurotoxicity, reversing neuromuscular paralysis, delaying or abrogating lethality, both immediately after envenoming and after onset of symptoms [25,28,29]. In combination, these studies have highlighted the great potential of varespladib as an orally available small molecule drug for use as a rapid snakebite intervention. 

Consequently, in this study we aimed to investigate which specific venom components can be inhibited by varespladib, with a focus on snake venoms that cause coagulopathic effects. Venoms from the medically relevant snake species *Bothrops asper*, *Calloselasma rhodostoma*, *Deinagkistrodon acutus*, *Daboia russelii*, *Echis carinatus*, *Echis ocellatus,* and *Oxyuranus scutellatus* were separated by liquid chromatography (LC) followed by high resolution fractionation (*nanofractionation*) onto 384-well plates allowing bioassaying of individual fractions for PLA_2_ and coagulation activities. Then, the potential inhibition of the detected activities by varespladib was evaluated and the toxins were identified by correlating parallel obtained mass spectrometry (MS) with proteomics data. Our findings show that varespladib is effective in inhibiting enzymatic activities of venom PLA_2_s as well as inhibiting coagulopathic toxins (of which many were tentatively identified as venom PLA_2_s).

## 2. Experimental Section

### 2.1. Chemicals

Water was purified using a Milli-Q Plus system (Millipore, Amsterdam, The Netherlands). Acetronitrile (ACN) (HPLC grade) and formic acid (FA) were purchased from Biosolve (Valkenswaard, The Netherlands). Calcium chloride (CaCl_2_, Dihydrate, ≥ 99%), NaCl, KCl, Tris base, Phosphate buffered saline (PBS) tablets, Triton X-100, L-*a*-Phosphatidylcholine, Varespladib (A-001, LY315920), and Cresol red were obtained from Sigma-Aldrich (Zwijndrecht, The Netherlands). Bovine plasma was obtained from Biowest (Nuaillé, France) and stored at −80 °C until use. Pooled venoms from *B. asper* (Costa Rica “Atlantic”), *C. rhodostoma* (captive bred, Thailand ancestry), *D. acutus* (captive bred, China ancestry), *D. russelii* (Sri Lanka), *E. carinatus* (India), *E. ocellatus* (Nigeria), and *O. scutellatus* (Papua New Guinea) were obtained from animals maintained in, or from the historical venom collection of, the Centre for Snakebite Research and Interventions, Liverpool School of Tropical Medicine (UK). These freeze-dried venoms were dissolved in water to a concentration of 5.0 ± 0.1 mg/mL and stored at −80 °C until use. PBS was prepared by dissolving PBS tablets in water according to the manufacturer’s instructions and stored at 4 °C for no longer than seven days. Varespladib was dissolved in DMSO (≥99.9%, Sigma-Aldrich, Zwijndrecht, The Netherlands) and stored at −20 °C. Prior to use, this varespladib stock solution was diluted in PBS to the required concentrations.

### 2.2. LC with Parallel Nanofractionation and MS Detection

Venom toxins were separated on a Shimadzu UPLC system (‘s Hertogenbosch, The Netherlands) which was controlled by Shimadzu Lab Solutions software. Venom solutions were diluted to 1.0 mg/mL in MilliQ water of which 50 μL was injected by a Shimadzu SIL-30AC autosampler. A Waters XBridge reverse-phase C18 column (250 × 4.6 mm column with a 3.5 μm pore size) was used under gradient elution at 30 °C. The temperature of the column was controlled by a Shimadzu CTO-30A column oven. By using two Shimadzu LC-30AD parallel pumps, the total solvent flow rate was maintained at 0.5 mL/min. Mobile phase A consisted of 98% H_2_O, 2% ACN, and 0.1% FA while mobile phase B was composed of 98% ACN, 2% H_2_O, and 0.1% FA. For gradient elution, mobile phase B was increased linearly from 0% to 50% in 20 min, then from 50% to 90% in 4 min. After reaching 90%, the flow rate of mobile phase B was kept at 90% for 5 min. For reconditioning, the mobile phase B was decreased from 90% to 0% in 1 min and kept at 0% for 10 min. The column effluent was split into two parts (9:1) of which the 10% fraction was sent to a UV detector (Shimadzu SPD-M20A Prominence diode array detector) while the remaining 90% was directed to a nanofraction collector. This was either a modified Gilson 235P autosampler programmed for nanofractionation and controlled by the in-house written software Ariadne, or a commercially available FractioMate^TM^ nanofractionator (SPARK-Holland and VU, Netherlands, Emmen and Amsterdam) controlled by the FractioMator software. Fractions were collected onto transparent 384-well plates (F-bottom, rounded square well, polystyrene, no lid, clear, non-sterile; Greiner Bio One, Alphen aan den Rijn, The Netherlands) at a resolution of 6 s/well. The plates with collected fractions were subsequently dried overnight using a Christ Rotational Vacuum Concentrator (RVC 2−33 CD plus, Zalm en Kipp, Breukelen, The Netherlands) equipped with a −80 °C cooling trap during the vacuum-drying process. The evaporated plates were stored at −20 °C until further use.

### 2.3. Phospholipase A_2_ Activity Assay

The PLA_2_ activity assay was carried out according to the method recently reported by Still et al. [30] using cresol red as a pH indicator. The PLA_2_ assay monitors the decrease in pH caused by the enzymatic conversion of L-*a*-Phosphatidylcholine to fatty acids. The assay solution was prepared freshly by dissolving NaCl (100 mM, final concentration), KCl (100 mM), CaCl_2_ (10 mM), Triton X-100 (0.875 mM), cresol red (0.02 mg/mL), and L-*a*-Phosphatidylcholine (0.875 mM) in a Tris buffer (1.0 mM, pH 8.0). The pH of the bioassay solution was checked prior to each run and adjusted to pH 8.0 by HCl if needed. For measurements, 40 μL of the assay solution was rapidly pipetted into each well of a vacuum-centrifuge-dried 384-well plate with venom fractions using a VWR Multichannel Electronic Pipette (10–200 μL; VWR International B.V., Amsterdam, The Netherlands) and a kinetic absorbance measurement at 572 nm was initiated immediately at room temperature using a plate reader (Varioskan™ Flash Multimode Reader, Thermo Fisher Scientific, Ermelo, The Netherlands). Kinetic measurements were collected over 40 min, and the PLA_2_ activity in each well was normalized by dividing the slope obtained for each well by the median of all the slope values obtained across the plate. For investigating PLA_2_ inhibition by varespladib, 10 μL aliquots of various concentrations varespladib (final concentrations of 20, 4, and 0.8 μΜ) were pipetted into each well of freeze-dried 384-well plates using a VWR Multichannel Electronic Pipette. Thereafter, the plates were centrifuged for 1 min at 805× *g* (2000 rpm) in a 5810 R centrifuge (Eppendorf, Germany) to remove potential air bubbles formed during the automated pipetting process, and then pre-incubated for 30 min at room temperature. Next, the PLA_2_ assay solutions were added as described above, and plate reader measurements were initiated. For comparison, 10 μL of PBS were added to each well and pre-incubated in the same manner as for the control experiments (indicated as PBS in the Figures). All analyses were performed in at least duplicate.

### 2.4. Plasma Coagulation Activity Assay

In-house aliquoted plasma was stored in 15 mL CentriStar^TM^ tubes (Corning Science, Reynosa, Mexico) at −80 °C. For preparing the aliquots, a 500 mL bottle of sodium citrate plasma (Sterile Filtered; Biowest, Nuaillé, France) stored at −80 °C was warmed in warm water until fully defrosted, after which the plasma was quickly aliquoted in 15 mL CentriStar^TM^ tubes, which were then immediately frozen at −80 °C, and stored until use. Prior to use, the 15 mL CentriStar^TM^ tubes were defrosted to room temperature in a warm water bath and then centrifuged at 805× *g* (2000 rpm) (Allegra^TM^ X-12 Centrifuge, Beckman Coulter) for 4 min to remove possible particulate matter.

For the coagulation assay, we followed our previously described approach [31,32]. Briefly, 20 μL CaCl_2_ solution (20 mM) was pipetted into each well of a freeze-dried plate using a Multidrop™ 384 Reagent Dispenser (Thermo Fisher Scientific, Ermelo, The Netherlands). This was followed by pipetting 20 μL of centrifuged plasma using the same Multidrop™ 384 Reagent Dispenser (after in-between rinsing the Multidrop with Milli-Q). Next, absorbance at 595 nm was monitored kinetically at room temperature on a plate reader (Varioskan™ Flash Multimode Reader, Thermo Fisher Scientific, Ermelo, The Netherlands). Measurements were collected over 100 min, and the slope of each well was normalized by dividing the slope measured in each well by the median of all slope values across the plate. The slope of the average 0–5 min reading was used for depicting very fast coagulation, whereas the slope of the average 0–20 min reading denoted slightly/medium increased coagulation. The slope of the single reading at 100 min was used to depict anticoagulation activity. Detailed explanations on the rationale of processing and plotting the data in this way are provided in [32,33].

To investigate whether varespladib was capable of inhibiting coagulopathic venom activity, 10 μL of various concentrations of the varespladib solution were added to each well of a freeze-dried 384-well plate (10 μL of PBS was added to the venom-only control). For all bioassay pipetting steps, a VWR Multichannel Electronic Pipette was used. The final concentrations of varespladib in the coagulation bioassay were 20, 4, and 0.8 μΜ (and in some cases also 0.16 and 0.032 μM). Directly after pipetting the varespladib solutions, plates were centrifuged for 1 min at 805× *g* (2000 rpm) using a 5810 R centrifuge (Eppendorf, Germany) and then pre-incubated for 30 min at room temperature. Meanwhile, the plasma coagulation activity assay solutions were prepared as described above and added to the plates after the pre-incubation step, after which the plates were measured on the plate reader. All analyses were performed in at least duplicate.

### 2.5. Correlation of Biological and MS Data

In our previous study [33], the same snake venoms as currently studied were analyzed using the nanofractionation approach, yielding accurate mass(es) of eluting venom toxins by MS and coagulopathic activities of fractions in parallel. In addition, proteomics data were acquired by an in-well tryptic digestion of the content of the wells that showed bioactivity followed by the LC-MS/MS analysis. The UniprotKB database was used to search for information on the class and possible known functions of relevant toxins. A correlation of the chromatographic LC-UV data acquired in this study with the previous study referred to above permitted the bioassay data generated in this study to be correlated with the MS and proteomics data previously obtained [33]. In order to identify potential molecular masses of bioactive toxins, firstly for each peak found in the bioassay trace, a mass spectrum was extracted by averaging the recorded spectra in the LC-MS trace over the corresponding time width at half maximum/minimum of the bioactive peak. Then, from all the detected ions in the average mass spectrum, extracted-ion chromatograms (XICs) were plotted. For XICs showing a peak shape and retention time matching to the bioactive peak under consideration, the corresponding *m*/*z* value was assigned to the bioactive compound. Finally, the deconvolution option in the MS software was used to determine the accurate monoisotopic masses of the bioactive compound.

## 3. Results and Discussion

In this study, a nanofractionation approach was used to evaluate the effects of varespladib on inhibiting PLA_2_ enzymatic activity and coagulopathic properties of individual venom toxins. After LC fractionation of venoms in 384-well plates, both the PLA_2_ enzymatic activities and the clotting activities of the individual venom fractions were evaluated. The inhibition of the measured venom toxin activities was assessed under different varespladib concentrations, and each active fraction detected was correlated with MS and proteomics data obtained in parallel to determine the identity of inhibited venom toxins.

### 3.1. PLA_2_ Bioactivity Profiles of Nanofractionated Venom Toxins

**Figure 1:**
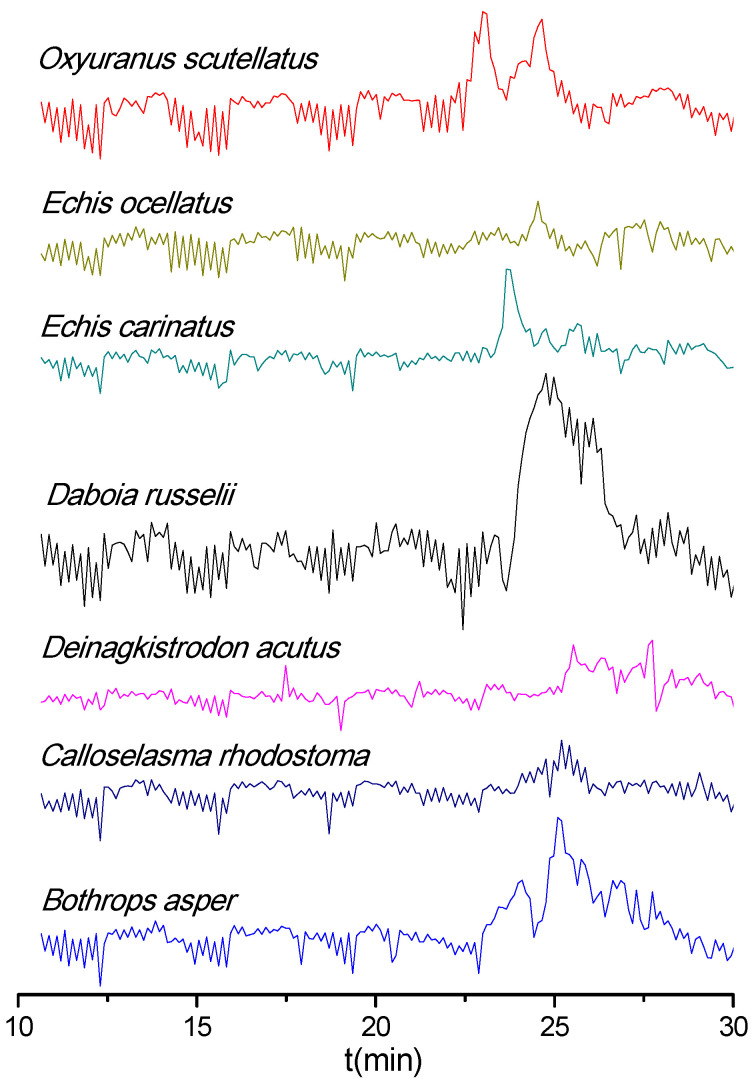
Phospholipase A_2_ (PLA_2_) bioactivity chromatograms of nanofractionated venom toxins. Positive peaks indicate PLA_2_ activity.

The PLA_2_ activity profiles of the snake venoms obtained after LC fractionation are shown as bioactivity chromatograms in Figure 1. Both *O. scutellatus* and *E. carinatus* venoms displayed relatively sharp peaks (two at 23.0 and 24.6 min for *O. scutellatus* and one at 23.6 min for *E. carinatus*). Conversely, *D. russelii* venom exhibited a broad and clear PLA_2_ activity peak (23.8–26.8 min), while *B. asper* displayed two closely eluting peaks (24.1 and 25.1 min) of which the first one (24.1 min) was observed close to the background level and the latter eluting peak (25.1 min) was distinctive and broad. For the other three venoms (*E. ocellatus*, *D. acutus,* and *C. rhodostoma*), no clear PLA_2_ bioactivity was observed at the analyzed venom concentration (1.0 mg/mL). All PLA_2_ bioactivity chromatograms resulting from duplicate measurements are presented in the Supplementary Materials (Section S1).

### 3.2. Coagulopathic Bioactivity Profiles of Nanofractionated Venom Toxins

**Figure 2:**
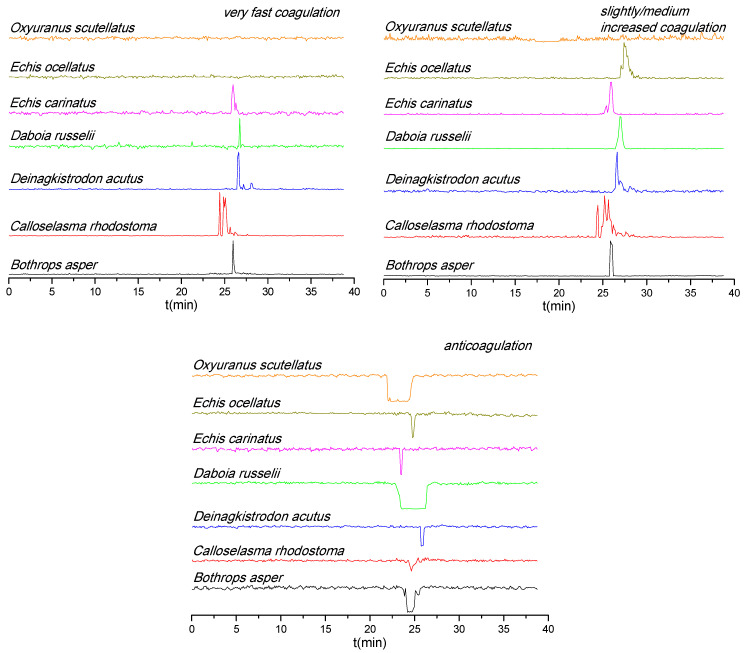
Coagulopathic bioactivity chromatograms of nanofractionated venom toxins. Anticoagulation is measured as negative signals and procoagulation as positive signals.

The coagulopathic bioactivities of nanofractionated venom components are shown in Figure 2. Most of the venoms displayed both pro and anticoagulant activities, except for the venom of *O. scutellatus*, for which only anticoagulant activity was observed. Note that during chromatographic separations under reversed-phase conditions non-stable toxin complexes and large toxins can denature, which could explain the lack of procoagulation observed for *O. scutellatus*, but a recent study demonstrated that higher venom concentrations were required to observe this effect with this venom after nanofractionation (i.e., 5.0 instead of 1.0 mg/mL) [33]. For both *D. russelii* and *O. scutellatus* venoms, the very broad anticoagulant peak observed indicates the presence of many closely eluting anticoagulant toxins—this activity was sufficiently potent to be observed visually on the plates after measurement. Among venoms with procoagulant activity, *E. ocellatus* venom only displayed a slightly/medium increased procoagulant activity, while very fast procoagulant activity was not observed. *C. rhodostoma* venom had a relatively weak anticoagulant and a strong procoagulant activity (for both very fast and slightly/medium increased coagulation). Note that despite the fact that in general venom toxins are rather stable, during RPLC within the nanofractionation analytics pipeline some venom toxins might have (partly) denatured and thereby lost their enzymatic activity. Bioactivity chromatograms of duplicate measurements and a detailed description of all observed coagulopathic peaks are shown in the Supplementary Materials (Section S2).

### 3.3. Neutralization Capabilities of Varespladib on the Enzymatic PLA_2_ Activity of Venom Toxins

**Figure 3:**
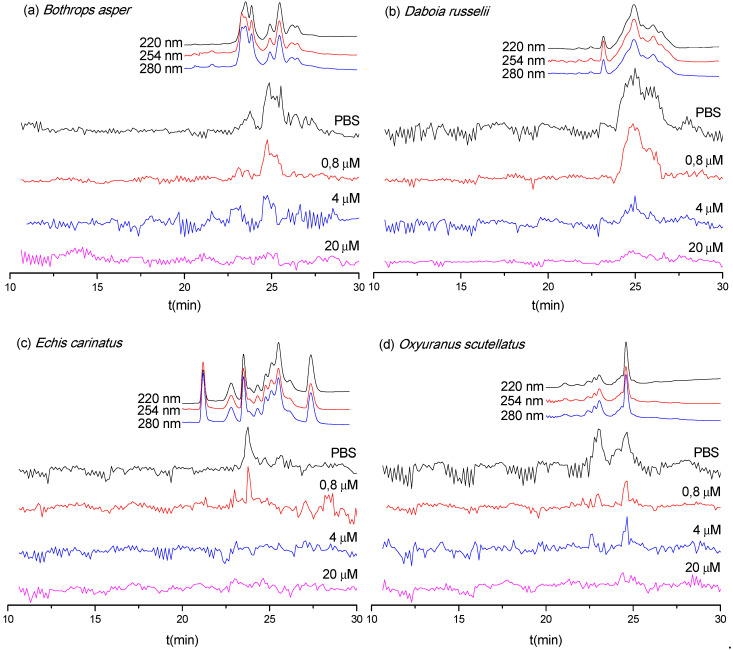
Superimposed PLA_2_ bioactivity chromatograms for nanofractionated venom toxins measured in the presence of the indicated concentrations of varespladib: (**a**) *B. asper*, (**b**) *D. russelii*, (**c**) *E. carinatus,* and (**d**) *O. scutellatus*. Top traces are the online LC-UV chromatograms recorded at 220, 254, and 280 nm for the respective venoms (allowing a correlation with LC-MS and proteomics data from Slagboom et al. [33]).

As discussed in Section 3.1, only LC fractions of the *B. asper*, *D. russelii*, *E. carinatus,* and *O. scutellatus* venoms were found to possess an abundantly detectable enzymatic activity in the PLA_2_ assay. Therefore, these four snake venoms were selected to assess the inhibitory effect of varespladib on the observed PLA_2_ activities of the fractions (Figure 3). As anticipated, the observed PLA_2_ activities for these four snake venoms decreased with increasing concentrations of varespladib. The PLA_2_ activities of *B. asper*, *D. russelii,* and *O. scutellatus* venoms were fully neutralized by 20 μM varespladib, whereas the activity observed for *E. carinatus* venom was abolished by 4 μM varespladib. These data indicate broad-spectrum venom PLA_2_ inhibition by varespladib. The duplicate bioassay chromatograms are presented in the Supplementary Materials (Section S3).

### 3.4. Neutralization Capabilities of Varespladib on Plasma Coagulation Activity of Venom Toxins

**Figure 4:**
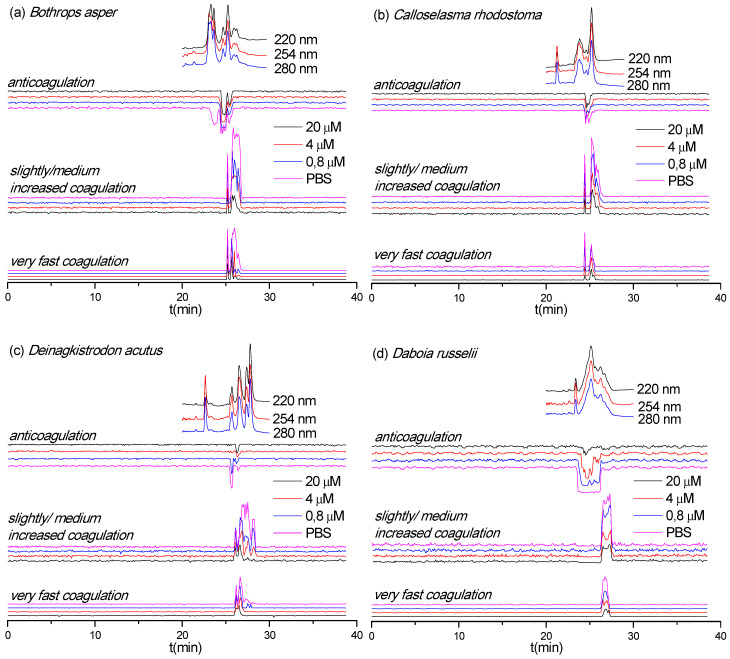

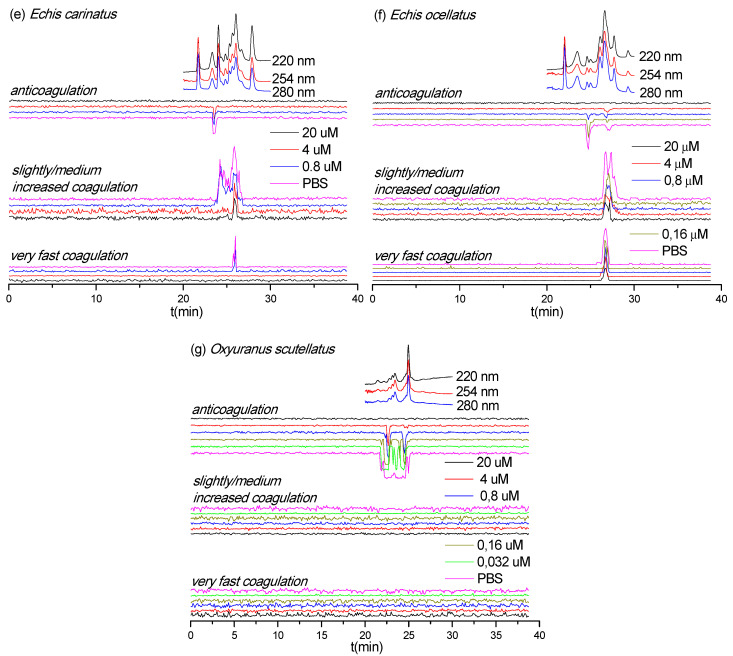
Coagulopathic toxicity chromatograms in the presence of various varespladib concentrations for nanofractionated venom toxins from (**a**) *B. asper*, (**b**) *C. rhodostoma*, (**c**) *D. acutus*, (**d**) *D. russelii*, (**e**) *E. carinatus*, (**f**) *E. ocellatus,* and (**g**) *O. scutellatus*. Top traces are the online LC-UV chromatograms recorded at 220, 254, and 280 nm for the respective venoms (allowing a correlation with LC-MS data and proteomics data from Slagboom et al. [33]).

Next, we assessed the inhibition of coagulopathic toxins identified in the various venom fractions by varespladib (Figure 4). Surprisingly, varespladib not only inhibited the anticoagulant activities of a number of the nanofractionated venom toxins, but also had an effect on some of the procoagulant venom fractions. Specifically, the anticoagulant activity of *E. carinatus*, *E. ocellatus,* and *O. scutellatus* venoms were fully neutralized by 20 μM varespladib. Varespladib was particularly effective in inhibiting the anticoagulant activity of toxins in *O. scutellatus* venom, and demonstrated a clear dose-response relationship. The anticoagulant activity of *D. acutus* and *D. russelii* venom components were almost completely abrogated with 20 μM varespladib, although trace activities remained. Contrastingly, varespladib did not considerably inhibit the anticoagulant toxicities observed in *B. asper* and *C. rhodostoma* venoms, with the exception that the first anticoagulant peak (23.1–24.2 min) in *B. asper* venom was fully inhibited at a very low concentration (0.8 μM varespladib). 

Varespladib also showed some inhibitory capabilities against the procoagulant activities of *B. asper*, *C. rhodostoma*, *D. acutus*, *D. russelii*, *E. carinatus,* and *E. ocellatus* venom (Figure 4). The extent of inhibition observed varied extensively, although full inhibition was not achieved across any of the venoms. The greatest effect was observed against the venom of *E. carinatus*, where the very fast coagulation activity was fully neutralized at 4 μM varespladib and most of the slightly/medium increased procoagulant activity was fully inhibited at 20 μM varespladib. The potent procoagulant activities of *D. russelii* venom were noticeably reduced in a dose-dependent manner, although full inhibition was not achieved, even when using the 20 μM varespladib concentration. Similar findings were observed with the venoms of *B. asper*, C. *rhodostoma*, *D. acutus,* and *E. ocellatus*, where procoagulant peaks were generally reduced in height with the highest concentrations of varespladib, suggesting perhaps a nonspecific inhibitory effect. The duplicate bioassay chromatograms and a detailed description of all coagulation-related activities neutralized by different concentrations of varespladib are provided in the Supplementary Materials (Section S4).

Snake venom PLA_2_s are well-known for their anticoagulant toxicities [34,35,36]. Our results show that varespladib effectively inhibits anticoagulant activities across a wide variety of medically important snake venoms. Additionally, we also find that varespladib can reduce the procoagulant venom activity, possibly by directly inhibiting enzymatic procoagulant toxins or blocking protein–protein interactions. However, the concentration of varespladib required to show noticeable inhibition of procoagulant venom activities was generally high (i.e., 20 μM), relative to that required for neutralizing anticoagulant activities.

### 3.5. Identification of Venom Toxins Neutralized by Varespladib

The correlated LC-MS (i.e., accurate masses of eluting venom toxins) and proteomics data obtained by Slagboom et al. [33] were used to identify venom toxins with enzymatic PLA_2_ and coagulopathic activities (Table 1 and Table 2). Bioactivities were linked to accurate molecular masses and tentative protein identities by aligning the characteristic LC-UV chromatograms obtained for each venom. When no exact mass data could be acquired by LC-MS, only the proteomics mass data obtained from the Mascot searches are provided.

Based on the results displayed in Table 1 and Figure 3, the PLA_2_ enzymes that were neutralized by varespladib could be tentatively identified. From the four species exhibiting enzymatic PLA_2_ activity after nanofractionation (i.e., *B. asper*, *E. carinatus*, *D. russelii,* and *O. scutellatus*) we detected a total of 13 toxins, of which all were unsurprisingly identified as PLA_2_ toxins. Eleven toxins were fully neutralized by 20 μM varespladib, while two were inhibited by much lower doses (0.8 μM varespladib). Variations were observed among the species, however, with five bioactive PLA_2_ enzymes identified in the venom of *B. asper*, four in *O. scutellatus*, three in *D. russelii,* and only one in *E. carinatus* (Table 1). 

The assigned toxins responsible for the coagulation activities observed are displayed in Table 2. Based on the data in Table 2 and Figure 4, the inhibitory potency of varespladib on the coagulopathic venom protein(s) was assessed. All tentatively identified anticoagulant toxins for the anticoagulant peaks from venoms of *B. asper*, *D. acutus*, *D. russelii*, *E. carinatus*, *E. ocellatus,* and *O. scutellatus* were fully abrogated by varespladib, while the anticoagulant toxins from *C. rhodostoma* were not inhibited by varespladib. No procoagulant toxins could be identified for the procoagulant peaks from the Mascot results for *D. russelii*, *E. carinatus,* and *O. scutellatus* venoms. Procoagulant toxins were identified from Mascot results for *B. asper*, *C. rhodostoma, D. acutus,* and *E. ocellatus* venoms, but we could not determine exactly which toxins were partially inhibited by varespladib as multiple venom toxins were found to co-eluted in each case. Thus, unambiguously assigning single toxins to each detected bioactivity is problematic at this resolution, especially if broad bioactivity peaks are observed. Additionally, when for example multiple potent anticoagulant toxins and a weak procoagulant toxin elute closely together, the net observed effect would be anticoagulation and the procoagulant toxin would not be detectable as a procoagulant. While distinction of all bioactive compounds in such cases requires further improving LC separations under toxin non-denaturating and MS compatible eluent conditions, it is worth noting that none of the tentatively assigned procoagulant toxins found here that were fully or partially inhibited by varespladib were PLA_2_s (see Table 2), suggesting varespladib may interact with other venom toxins. A detailed description of the results discussed here is provided in the Supplementary Materials (Section S5). 

## 4. Conclusions

A recently developed analytical platform combining LC, MS, and PLA_2_ and coagulation activity bioassays was applied to evaluate the inhibitory properties of varespladib against the enzymatic PLA_2_ and coagulopathic activities of toxins found in the venoms of several medically important snake species. All venoms analyzed in this study showed constituents with clear coagulopathic toxicities, while only the venoms of *B. asper*, *D. russelii*, *E. carinatus,* and *O. scutellatus* displayed components with a clear enzymatic PLA_2_ activity. All components with detected enzymatic PLA_2_ activities were identified as PLA_2_ toxins and were fully neutralized by the small molecule toxin inhibitor varespladib. We demonstrated here that varespladib inhibited many of the anticoagulant bioactivities of the toxin components found in these venoms, similar to findings recently described by others for certain snakes of the genera *Naja*, *Pseudechis* and *Bitis* [16,37,38], and we confirmed that the toxins responsible are likely to be PLA_2_s based on correlations between MS and proteomics data and the bioactivity chromatograms. However, we also revealed that several of the procoagulant venom toxins were also neutralized to some degree by varespladib. These findings suggest that the mechanism underlying venom inhibition may not be solely based on inhibition of the active site of venom PLA_2_s, as other toxin types are typically responsible for procoagulant venom activities. However, we cannot rule out that nonspecific effects at high inhibitor concentrations are responsible for these observations, and thus future work is required to robustly explore this. Note that during chromatographic separations under reversed-phase conditions nonstable toxin complexes and large toxins can denature. Currently, we cannot circumvent this potential drawback of the nanofractionation analytics. Overall, our data further support the value of varespladib as a potential new therapeutic for mitigating the toxic effects of certain snakebites [9], and they re-emphasize that while this small molecule toxin inhibitor is a highly promising treatment for combatting neurotoxicity [25,28,29], it may also be of great value for treating elements of hemotoxicity caused by snake envenoming.

## Figures and Tables

**Table 1:** Correlated accurate molecular masses and proteomics data for PLA_2_ activities (peak retention times are adapted from Figure 3).

Species	Peaks Retention Time (min)	Mascot Results Matching the Exact Mass	*m/z* Values from MS Data	Exact Mass from MS Data	Exact Mass Calculated from Mascot Data	Toxin Class	Varespladib Concentration Required for Full Inhibition
*B* *. asper*	23.2–24.1	PA2H2_BOTAS	1373.3688^10+^	13,714.5646	13,714.56817	PLA_2_	0.8 μM
	24.3–25.8	PA2HA_BOTAS	1266.5985^11+^	13,912.4649	13,896.51308	PLA_2_	20 μM
	24.3–25.8	PA2H3_BOTAS	1378.3697^10+^	13,765.5812	13,765.58896	PLA_2_	20 μM
	24.3–25.8	PA2B3_BOTAS	1164.8811^12+^	13,957.5333	13,957.48720	PLA_2_	20 μM
	24.3–25.8	PA2A2_BOTAS	-	-	14,194	PLA_2_	20 μM
*D. russelii*	23.9–27.4	PA2B8_DABRR	1511.6962^9+^	13,587.2248	13,587.2027	PLA_2_	20 μM
	23.9–27.4	PA2B5_DABRR			13,587	PLA_2_	20 μM
	23.9–27.4	PA2B3_DABRR	-	-	13,687	PLA_2_	20 μM
*E. carinatus*							
	23.3–24.4	PA2A1_ECHCA	-	-	16,310	PLA_2_	0.8 μM
						
*O. scutellatus*	22.6–25.1	PA2TA_OXYSC	-	-	13,829	PLA_2_	20 μM
	22.6–25.1	PA2TB_OXYSC	-	-	16,008	PLA_2_	20 μM
	23.6–25.1	PA21_OXYSC	-	-	16,898	PLA_2_	20 μM
	23.6–25.1	PA2TC_OXYSC	-	-	13,313	PLA_2_	20 μM

**Table 2:** Correlated LC-MS masses and proteomics data for coagulopathic venom toxins activities (peak retention times are adapted from Figure 4; SVMP: Snake Venom Metalloproteinase; SVSP: Snake Venom Serine Protease; CTL: C-Type Lectin; kunitz: kunitz-type serine protease inhibitor; PN: Partly Neutralized at 20 μM varespladib; NOI: No Observed Inhibition.

Species	Peak Retention Time (min)	Peak Activity	Mascot Results Matching the Exact Mass	*m/z* Values from MS Data	Exact Mass from MS Data	Exact Mass Calculated from Mascot Data	Toxin Class	Varespladib Concentration Needed for Full Inhibition
*B* *. asper*	23.1–24.2	Anticoagulation	PA2H2_BOTAS	1373.3688^10+^	13,714.5646	13,714.56817	PLA_2_	0.8 μM
	24.2–25.2	Anticoagulation	PA2HA_BOTAS	1266.5985^11+^	13,912.4649	13,896.51308	PLA_2_	20 μM
	24.2–25.2	Anticoagulation	PA2H3_BOTAS	1378.3697^10+^	13,765.5812	13,765.58896	PLA_2_	20 μM
	25.2–25.8	Anticoagulation	PA2B3_BOTAS	1164.8811^12+^	13,957.5333	13,957.48720	PLA_2_	20 μM
	25.2–25.8	Anticoagulation	PA2A2_BOTAS	–	-	14,194	PLA_2_	20 μM
	25.2–25.8	Anticoagulation	VM2_BOTAS	-	-	53,564	SVMP	NOI
	25.0–26.8	Procoagulation	VSPL_BOTAS	-	-	28,019	SVSP	PN
	25.0–26.8	Procoagulation	VM1B1_BOTAS	-	-	45,936	SVMP	PN
	25.4–26.8	Procoagulation	SLA_BOTAS	-		7084	CTL	PN
*C* *. rhodostoma*	24.3–25.5	Anticoagulation	PA2BD_CALRH	1244.1103^11+^	13,665.0848	13,665.0237	PLA_2_	NOI
	24.3–25.5	Anticoagulation	PA2AB_CALRH	-	-	14,352	PLA_2_	NOI
	24.3–25.5	Anticoagulation	VSPF1_CALRH	-	-	26,570	SVSP	NOI
	24.3–25.5	Anticoagulation	SLEA_CALRH	-	-	15,962	CTL	NOI
	24.3–25.5	Anticoagulation	SLEB_CALRH	-	-	15,190	CTL	NOI
	24.3–26.6	Procoagulation	VSPF2_CALRH	-	-	29,145	SVSP	PN
	24.9–26.6	Procoagulation	SLYA_CALRH	-	-	15,796	CTL	PN
	24.9–26.6	Procoagulation	SLYB_CALRH	-	-	16,770	CTL	PN
*D* *. acutus*	25.4–25.9	Anticoagulation	PA2A_DEIAC	-	-	14,820	PLA_2_	4 μM
	25.4–25.9	Anticoagulation	SL_DEIAC	-	-	18,332	CTL	4 μM
	26.0–27.2	Procoagulation	VSP1_DEIAC	-	-	29,480	SVSP	PN
	26.0–27.2	Procoagulation	VSPA_DEIAC	-	-	26,132	SVSP	PN
	26.4–27.8	Procoagulation	SLCB_DEIAC	-	-	17,133	CTL	PN
	26.4–27.8	Procoagulation	VM1AC_DEIAC	-	-	47,690	SVMP	PN
	26.4–27.8	Procoagulation	VM11_DEIAC	-	-	47,845	SVMP	PN
	26.4–27.8	Procoagulation	VM1H5_DEIAC	-	-	46,518	SVMP	PN
	26.4–27.8	Procoagulation	VM3AK_DEIAC	-	-	69,752	SVMP	PN
	27.8–28.4	Procoagulation	VM3A2_DEIAC	-		27,151	SVMP	20 μM
	27.8–28.4	Procoagulation	VM3AH_DEIAC	-		70,721	SVMP	20 μM
*D. russelii*	23.4–26.4	Anticoagulation	PA2B8_DABRR	1511.6962^9^^+^	13,587.2248	13,587.2027	PLA_2_	20 μM
	23.4–26.4	Anticoagulation	PA2B5_DABRR	–		13,587	PLA_2_	20 μM
	23.4–26.4	Anticoagulation	PA2B3_DABRR	–		13,687	PLA_2_	20 μM
	26.2–27.6	Procoagulation	–	-	-	-	-	
*E. carinatus*	23.3–23.8	Anticoagulation	PA2A1_ECHCA	-	-	16,310	PLA_2_	0.8 μM
	23.8–26.9	Procoagulation	-	-	-	-	-	
							
*E. ocellatus*	24.4–25.1	Anticoagulation	PA2A5_ECHOC	1541.4718^9+^	13,856.1382	13,856.0665	PLA_2_	4 μM
	26.3–28.2	Procoagulation	VM3E2_ECHOC	-	-	69,426	SVMP	PN
	26.3–28.2	Procoagulation	VM3E6_ECHOC	-	-	57,658	SVMP	PN
	26.3–28.2	Procoagulation	SL1_ECHOC	-	-	16,601	CTL	PN
	26.3–28.2	Procoagulation	SL124_ECHOC	-	-	16,882	CTL	PN
*O. scutellatus*	21.7–25.2	Anticoagulation	PA2TA_OXYSC	-	-	13,829	PLA_2_	20 μM
	21.7–25.2	Anticoagulation	PA2TB_OXYSC	-	-	16,008	PLA_2_	20 μM
	21.7–25.2	Anticoagulation	PA21_OXYSC	-	-	16,898	PLA_2_	20 μM
	21.7–25.2	Anticoagulation	PA2TC_OXYSC	-	-	13,313	PLA_2_	20 μM
	21.7–25.2	Anticoagulation	VKT_OXYSC		-	9711	kunitz	20 μM
	21.7–25.2	Anticoagulation	VKT3_OXYSC	-	-	9029	kunitz	20 μM

## Supplementary Materials

The following are available online at https://www.mdpi.com/2227-9059/8/6/165/s1, Figure S1. Duplicate PLA_2_ bioactivity chromatograms of nanofractionated venom toxins, positive peaks indicate PLA_2_ activity; Figure S2. Duplicate coagulopathic toxicity chromatograms of the nanofractionated venom toxins, anticoagulation is measured as negative signals and procoagulation as positive signals; Figure S3. Duplicate PLA_2_ bioactivity chromatograms of *B. asper*, *D. russelii*, *E. carinatus,* and *O. scutellatus* venoms in the presence of various varespladib concentrations; Figure S4. Duplicate coagulopathic toxicity bioassay chromatograms of nanofractionated venom toxins from *B. asper, C. rhodostoma, D. acutus, D. russelii, E. carinatus, E. ocellatus,* and *O. scutellatus* in the presence of various concentrations of varespladib.
