# Supplementary material for: Varespladib Inhibits the Phospholipase A2 and Coagulopathic Activities of Venom Components from Hemotoxic Snakes
Source: Biomedicines. 2020 Jun 17;8(6):165. doi: 10.3390/biomedicines8060165 (PMC7345350; doi:10.3390/biomedicines8060165)
Supplement: Supplementary file 1 [file biomedicines-08-00165-s001.zip › biomedicines-814884-supplementary.docx]

Supporting Information

Varespladib Inhibits the Phospholipase A_2_ and Coagulopathic Activities of Venom Components from Hemotoxic Snakes

Chunfang Xie, Laura-Oana Albulescu, Kristina B. M. Still, Julien Slagboom, Yumei Zhao, Zhengjin Jiang, Govert W. Somsen, Freek J. Vonk, Nicholas R. Casewell and Jeroen Kool

S1. Duplicate PLA_2_ bioactivity chromatograms of nanofractionated venom toxins

The PLA_2_ bioactivity chromatograms of the individual venom toxins from *Bothrops asper*, *Calloselasma rhodostoma*, *Deinagkistrodon acutus*, *Daboia russelii*, *Echis carinatus*, *Echis ocellatus* and *Oxyuranus scutellatus* after nanofractionation at 1.0 mg/ml are shown in Figure S1. In this study, each measurement was performed at least in duplicate following nanofractionation for assessing repeatability.

**Figure S1.** Duplicate PLA_2_ bioactivity chromatograms of nanofractionated venom toxins. Positive peaks indicate PLA_2_ activity.

S2. Coagulopathic pathologies of nanofractionated venom toxins

The duplicate coagulation bioassay chromatograms are shown in Figure S2. Each venom was nanofractionated at 1.0 mg/ml. For *B. asper* venom, a sharp potent positive peak was observed in both the very fast and the slightly/medium increased coagulation chromatograms, whereas a broad potent negative peak was seen in the anticoagulation chromatogram. For *C. rhodostoma* venom, a sharp potent positive peak, followed by several potent none-baseline positive peaks was observed in both the very fast and the slightly/medium increased coagulation chromatograms, while only a relatively weak negative peak present in anticoagulation chromatogram. For both *D. acutus* and *E. carinatus* venoms, a sharp potent positive peak was noted in the very fast and the slightly/medium increased coagulation chromatograms, while a sharp potent negative peak was observed in the anticoagulation chromatograms. For *D. russelii* venom, a sharp potent positive peak was observed in both the very fast and the slightly/medium increased coagulation chromatograms. A relatively broad and potent negative peak, which was generated by several coagulopathic proteins eluting closely together, was observed in the anticoagulation chromatogram. For *E. ocellatus* venom, a sharp potent peak was observed in both the slightly/medium increased procoagulation and the anticoagulation chromatograms, while no activity was observed in the very fast coagulation chromatogram. For *O. scutellatus* venom, a clear broad anticoagulant peak was observed, similar to that in *D. russelii* venom. However, no procoagulant activity was observed with *O. scutellatus* venom at the tested concentration of 1.0 mg/ml.

**Figure S2.** Duplicate coagulopathic toxicity chromatograms of the nanofractionated venom toxins. Anticoagulation is measured as negative signals and procoagulation as positive signals. *(figure continues on next page).*

**Figure S2.** *(continuation of figure from previous page).* Duplicate coagulopathic toxicity chromatograms of the nanofractionated venom toxins. Anticoagulation is measured as negative signals and procoagulation as positive signals.

S3. Duplicate chromatograms displaying the inhibitory effects of varespladib on enzymatic PLA_2_ activity.

Duplicate PLA_2_ bioactivity chromatograms showing the ability of varespladib to neutralize nanofractionated venom proteins from *B. asper*, *D. russelii*, *E. carinatus* and *O. scutellatus* are depicted in Figure S3. In each figure, the bioassay chromatograms depicted on the right side (which do not have superimposed correlated UV data) represent replicates of the chromatograms on the left side (which are also presented in the main manuscript).

**Figure S3.** Duplicate PLA_2_ bioactivity chromatograms of *B. asper*, *D. russelii*, *E. carinatus* and *O. scutellatus* venoms in presence of various varespladib concentrations. *(figure continues on next page).*

**Figure S3.** *(continuation of figure from previous page).* Duplicate PLA_2_ bioactivity chromatograms of *B. asper*, *D. russelii*, *E. carinatus* and *O. scutellatus* venoms in presence of various varespladib concentrations.

S4. Duplicate chromatograms displaying the inhibitory effects of varespladib on coagulopathic venom toxins.

The inhibitory capabilities of varespladib against the nanofractionated venom proteins involved in modulating plasma coagulation for *B. asper*, *C. rhodostoma*, *D. acutus*, *D. russelii*, *E. carinatus*, *E. ocellatus* and *O. scutellatus* venoms was assessed after nanofractionation of 1.0 mg/ml venom concentrations. The duplicate coagulation bioassay chromatograms are shown in Figure S4. For all venoms, varespladib was assayed at concentrations of 20 μM, 4 μM and 0.8 μM. Where the 0.8 μM varespladib concentration still inhibited the coagulopathic venom components, lower concentrations were additionally evaluated (i.e. 0.16 μM and 0.032 μM if needed).

In the venom-only chromatograms for *B. asper* venom, a sharp positive peak together with a broad none-baseline co-eluting positive peak were identified both for the very fast and the slightly/medium increased coagulation activities, whereas a broad negative peak followed by a series of potent co-eluting negative peaks ending in a relatively sharp negative peak could be observed for anticoagulant activity. In the presence of varespladib, all procoagulant activities were inhibited with peaks becoming sharper in a concentration-dependent manner. The tailing peak in the very fast coagulation chromatogram was effectively neutralized by 4 μM varespladib, whereas the two front-eluting peaks were still visible. At the highest varespladib concentration, the peaks were further reduced in intensity, but could not be fully neutralized. For the slightly/medium increased coagulation activity, both peaks were reduced dose-dependently by varespladib, but moderate activity was still observed at 20 μM varespladib. For the anticoagulant activity profile, the front broad negative peak was only visible for the venom-only measurements, suggesting that varespladib could effectively inhibit the eluted venom protein(s). In contrast, the other activity peaks were not affected by varespladib, demonstrating that these toxins could not be inhibited by varespladib.

For *C. rhodostoma* venom, two clear peaks were noted in the very fast procoagulation activity chromatogram, which were equally reduced in intensity by increasing varespladib concentrations, but to a limited extent, as none could be fully neutralized by 20 μM varespladib. The same was observed for the slightly/medium increased coagulation activity. Here, the second (largest) peak consisted of three closely co-eluting peaks, of which the third peak was largely neutralized by 20 μM varespladib. The other two peaks were also partially neutralized, but to a lesser extent. Two peaks can clearly be seen in the anticoagulant activity chromatogram and were not significantly affected by varespladib.

For *D. acutus* venom, both the very fast coagulation and the slightly/medium increased coagulation activities resulted in multiple co-eluting chromatographic peaks. The intensity of these peaks decreased with increasing varespladib concentrations, suggesting inhibition of most procoagulant proteins in this venom. The front two peaks in the procoagulation bioactivity chromatograms alone could not be fully inhibited by 20 μM varespladib. Moreover, one potent sharp negative peak and a much smaller later eluting negative peak could be observed in the anticoagulation chromatograms. The intensity of the sharp negative front peak decreased in a concentration-dependent manner in the presence of varespladib, and was fully neutralized by 20 μM varespladib, while the later eluting weak negative peak was unaffected by varespladib.

For *D. russelii* venom, a strong and broad peak in the very fast procoagulant profile was visible, while several overlapping procoagulant peaks were noted in the slightly/medium increased coagulation chromatograms in the venom-only analysis. In the anticoagulation profile, one very broad anticoagulation signal (which also can be seen by visually inspecting a plate after the measurement, as the respective wells did not show any plasma coagulation) was observed in the venom-only analysis. This negative broad peak indicates many closely eluting proteins. With increasing varespladib concentrations, the signal for the very fast procoagulant activity decreased in a similar manner as the slightly/medium increased coagulation activity. In both bioactivity chromatograms, the broad peak profiles were split into two non-baseline peak areas, representing multiple eluting procoagulant proteins. A clear dose-response effect was observed, but varespladib could not fully inhibit procoagulant activity at the highest concentration tested (20 μM). The very broad and potent negative peak in the anticoagulation chromatogram was drastically reduced and narrowed until only a weak peak was retained. This indicated the efficient neutralization of anticoagulant activity by varespladib.

For *E. carinatus* venom, a sharp peak was observed in the very fast coagulation activity chromatogram in the venom-only analysis, which was neutralized by 4 μM varespladib. The slightly/medium increased procoagulant activity displayed two areas of potent broad positive peaks in the venom-only analysis, of which the front area of peaks was efficiently neutralized at the low varespladib concentrations of 4 μM. The later eluting peaks were only partially inhibited by varespladib and one of these decreased in intensity in a concentration-dependent fashion; however, a full inactivation of this peak was not achieved even at 20 μM varespladib. In the anticoagulation chromatogram, one sharp negative peak was detected and was reduced by varespladib in a dose-dependent manner; full neutralization was observed at 20 μM varespladib.

For *E. ocellatus* venom, a very weak positive peak followed by a potent sharp positive peak was observed in the very fast coagulation chromatogram in the venom-only analysis. The front weak positive peak was fully neutralized by varespladib at the very low concentration of 0.16 μM. The activity of the strong positive peak decreased with increasing concentrations of varespladib, despite full inactivation not being achieved even at 20 μM varespladib. The slightly/medium increased coagulation chromatogram displayed two sharp positive non-baseline peaks. Varespladib also inhibited this positive shoulder peak in a concentration-dependent manner, while full inactivation was not achieved at 20 μM varespladib. In contrast to the procoagulant profile, the anticoagulation activity profile was effectively neutralized by varespladib. A potent sharp negative peak followed by a later eluting weak negative peak was observed in the venom-only chromatogram. The front potent sharp negative peak decreased with increasing concentrations of varespladib, and some residual activity was observed at a 0.8 μM varespladib. No activity was detected at 4 μM varespladib. The later eluting weak negative peak was not influenced by varespladib at low concentrations and only was fully neutralized by 20 μM varespladib.

For *O. scutellatus* venom, no activity was detected for both the very fast coagulation and the slightly/medium increased coagulation chromatograms. A moderate negative peak followed by a very broad and strong negative peak and finally a sharp negative peak were observed in the anticoagulation chromatogram for the venom-only analysis. At the very low varespladib concentration of 0.032 μM, the effective inhibition of anticoagulant activity was observed. The main broad negative peak was reduced into four individual negative peaks, which were observed as a broad negative peak followed by two sharp negative peaks and one additional broad negative peak. The latest eluting sharp negative peak observed in the venom-only chromatogram was fully neutralized by 0.032 μM varespladib. By further increasing the varespladib concentration to 0.16 μM, the four individual negative peaks observed in the 0.032 μM varespladib analysis decreased further in both peak broadness and height, and the middle two sharp negative peaks were neutralized. The two potent negative left peaks were effectively decreased by further increasing varespladib concentrations; of which the front eluting negative peak was fully neutralized by 20 μM varespladib and the later eluting negative peak was fully neutralized by 4 μM varespladib. No activity was detected at a varespladib concentration of 20 μM.

**Figure S4.** Duplicate coagulopathic toxicity bioassay chromatograms of nanofractionated venom toxins from *B. asper*, *C. rhodostoma*, *D. acutus*, *D. russelii*, *E. carinatus*, *E. ocellatus* and *O. scutellatus* in the presence of various concentrations of varespladib. *(figure continues on next page).*

**Figure S4.** *(continuation of figure from previous page)*. Duplicate coagulopathic toxicity bioassay chromatograms of nanofractionated venom toxins from *B. asper*, *C. rhodostoma*, *D. acutus*, *D. russelii*, *E. carinatus*, *E. ocellatus* and *O. scutellatus* in the presence of various concentrations of varespladib.

**Figure S4.** *(continuation of figure from previous page)*. Duplicate coagulopathic toxicity bioassay chromatograms of nanofractionated venom toxins from *B. asper*, *C. rhodostoma*, *D. acutus*, *D. russelii*, *E. carinatus*, *E. ocellatus* and *O. scutellatus* in the presence of various concentrations of varespladib.

S5. Inhibitory efficacy of varespladib on venom toxins identified in Table 2.

The anticoagulant PA2H2_BOTAS was fully neutralized by 0.8 μM varespladib and the anticoagulants PA2A_DEIAC and SL_DEIAC were fully neutralized by 4 μM varespladib. The anticoagulants PA2B8_DABRR, VKT_OXYSC and VKT3_OXYSC were fully neutralized by 20 μM varespladib. The pro-coagulants VM3A2_DEIAC and VM3AH_DEIAC were neutralized by 20 μM varespladib. In contrast, the anticoagulants VM2_BOTAS, PA2AB_CALRH, SLEA_CALRH, SLEB_CALRH and VSPF1_CALRH were not inhibited by varespladib. Moreover, the pro-coagulants VSPL_BOTAS, VM1B1_BOTAS, SLA_BOTAS, VSPF2_CALRH, SLYA_CALRH, SLYB_CALRH, VSP1_DEIAC, VSPA_DEIAC, SLCB_DEIAC, VM1AC_DEIAC, VM11_DEIAC, VM1H5_DEIAC, VM3AK_DEIAC, VM3E2_ECHOC, VM3E6_ECHOC, SL1_ECHOC and SL124_ECHOC were matched to the time frame of peaks which were moderately inhibited by varespladib. Further investigation is needed to decide whether they can be neutralized by varespladib.

Of the identified PLA_2_ enzymes, no anticoagulant activity was reported in UniprotKB for PA2HA_BOTAS, PA2H3_BOTAS, PA2B3_BOTAS, PA2A2_BOTAS, PA2BD_CALRH, PA2B3_DABRR, PA2B5_DABRR, PA2A1_ECHCA, PA2A5_ECHOC, PA2TA_OXYSC, PA2TB_OXYSC, PA21_OXYSC and PA2TC_OXYSC. Of these toxins, PA2A1_ECHCA was fully neutralized by 0.8 μM varespladib, PA2A5_ECHOC was fully neutralized by 4 μM varespladib, PA2HA_BOTAS, PA2H3_BOTAS, PA2B3_BOTAS, PA2A2_BOTAS, PA2B3_DABRR, PA2B5_DABRR, PA2TA_OXYSC, PA2TB_OXYSC, PA21_OXYSC and PA2TC_OXYSC were fully inhibited by 20 μM varespladib. PA2BD_CALRH was not inhibited by varespladib. PA2B3_BOTAS was reported to show myotoxic activity [[1](#_ENREF_1)] and PA2A2_BOTAS to catalyze the Ca^2+^-dependent hydrolysis of 2-acyl groups in 3-sn-phosphoglycerides [[2](#_ENREF_2)]. PA2BD_CALRH is known to be enzymatically inactive because of the evolutionary loss of its calcium binding site [[3](#_ENREF_3)]. PA2B5_DABRR was reported to catalyze the Ca^2+^-dependent hydrolysis of the 2-acyl groups in 3-sn-phosphoglycerides [[4](#_ENREF_4),[5](#_ENREF_5)]. PA2TA_OXYSC was reported as neurotoxic and capable of catalyzing the Ca^2+^-dependent hydrolysis of 2-acyl groups in 3-sn-phosphoglycerides [[6](#_ENREF_6)]. PA2TB_OXYSC was also reported to possess neurotoxic properties [[7](#_ENREF_7)].

References

1. Kaiser, I.I.; Gutiérrez, J.M.; Plummer, D.; Aird, S.D.; Odell, G.V. The amino acid sequence of a myotoxic phospholipase from the venom of *Bothrops asper*. *Archives of biochemistry and biophysics* **1990**, *278*, 319-325.

2. Fernández, J.; Gutiérrez, J.M.; Angulo, Y.; Sanz, L.; Juárez, P.; Calvete, J.J.; Lomonte, B. Isolation of an acidic phospholipase A_2_ from the venom of the snake *Bothrops asper* of Costa Rica: biochemical and toxicological characterization. *Biochimie* **2010**, *92*, 273-283.

3. Tsai, I.H.; Wang, Y.M.; Au, L.C.; Ko, T.P.; Chen, Y.H.; Chu, Y.F. Phospholipases A_2_ from *Callosellasma rhodostoma* venom gland: Cloning and sequencing of 10 of the cDNAs, three‐dimensional modelling and chemical modification of the major isozyme. *European journal of biochemistry* **2000**, *267*, 6684-6691.

4. Tsai, I.-H.; Lu, P.-J.; Su, J.-C. Two types of *Russell's viper* revealed by variation in phospholipases A_2_ from venom of the subspecies. *Toxicon* **1996**, *34*, 99-109.

5. Suzuki, M.; Itoh, T.; BM, A.; Athauda, S.B.; Moriyama, A. Molecular diversity in venom proteins of the Russell's viper (*Daboia russellii russellii*) and the Indian cobra (*Naja naja*) in Sri Lanka. *Biomedical Research* **2010**, *31*, 71-81.

6. Cendron, L.; Mičetić, I.; Polverino de Laureto, P.; Paoli, M. Structural analysis of trimeric phospholipase A_2_ neurotoxin from the Australian taipan snake venom. *The FEBS journal* **2012**, *279*, 3121-3135.

7. Fohlman, J.; eaker, D.; karlsson, E.; thesleff, S. Taipoxin, an extremely potent presynaptic neurotoxin from the venom of the Australian snake taipan (*Oxyuranus s. scutellatus*) Isolation, characterization, quaternary structure and pharmacological properties. *European journal of biochemistry* **1976**, *68*, 457-469.
